# Exploring perceived access to and previous experiences with general practice and associations with health literacy in the Danish population

**DOI:** 10.1080/02813432.2025.2583706

**Published:** 2025-11-24

**Authors:** Lisa Maria Sele Sætre, Ditte Krag-Hansen, Jens Søndergaard, Dorte Ejg Jarbøl, Kirubakaran Balasubramaniam

**Affiliations:** Department of Public Health, Research Unit of General Practice, University of Southern Denmark, Odense M, Denmark

**Keywords:** General practice, primary health care, healthcare seeking, accessibility, vulnerable groups, equity

## Abstract

**Aim:**

To (1) explore perceived access to general practice, relationship with the general practitioner (GP) and previous experiences with general practice among the Danish population and (2) analyse the associations with sex, age, chronic disease and health literacy.

**Methods:**

A cross-sectional nationwide survey study among 100,000 randomly selected adults aged 20 years or above. Questionnaire data comprised items covering the perceived access to, relationship and previous experiences with GP contacts, chronic disease and health literacy. Data were linked to register data. Descriptive statistics and multivariable logistic regression models were applied.

**Results:**

A total of 27,713 (30%) individuals were included. More than a third reported difficulties with talking to the GP secretary (35%) and with getting an appointment with their preferred doctor (44%). Some 80% reported high confidence in the GP, whereas previous negative experiences and insufficient consultation time were reported by 33% and 46%, respectively. Females and individuals with health literacy challenges in terms of being less able to actively engage with healthcare professionals were more likely to report difficulties with access to and previous negative experiences with general practice. Individuals with higher age, chronic disease(s) and health literacy challenges in terms of feeling less understood and supported, and less ability to actively engage with healthcare providers were less confident in their GP.

**Conclusions:**

This study highlights difficulties related to accessing general practice and previous negative experiences among different population groups. Since some individuals are more likely to encounter these challenges, differentiating healthcare services may promote greater equity in health.

## Background

In Denmark, most clinical evaluations and diagnoses of both acute and chronic illnesses, as well as monitoring of existing diseases, are managed in general practice [1]. Contacting the healthcare system can be challenging for some people. Several barriers to healthcare seeking exist, such as fear of being diagnosed with a severe illness and uncertainties about how to navigate the healthcare system [2]. Being a patient can be daunting and overwhelming, especially for those who are less familiar with the organisation of the healthcare system or who have had negative experiences in previous encounters with healthcare professionals [3,4].

The capacity, personal competencies and situational resources needed for people to access, understand, appraise and use information and services to make decisions about their health are included in the definition of health literacy [5]. Studies have shown that health literacy is a social determinant of inequity in public health [6–8], acute hospital admissions and re-admissions [9], diagnostic courses [10], as well as prognoses for several conditions [11,12]. Some of the inequity in healthcare may arise before individuals enter the healthcare system, not only due to risk behaviours or genetics, but also due to healthcare-seeking behaviour [13,14]. Making the decision to seek care is complex and can be affected by chronic disease, personal relations and concerns [15,16]. These factors can be related to an interplay of biological and psychological factors as well as social and cultural norms [17], but the organisation of the healthcare system, including general practice, may also be of importance for the healthcare-seeking behaviour [18].

Despite claims of free and equal access to healthcare in Denmark, disparities in healthcare seeking, diagnostics and prognoses exist for several diseases [19]. The Danish healthcare system is universal and primarily financed by taxation. More than 98% of all citizens are listed with a general practice, and general practitioners (GPs) serve as gatekeepers who can refer patients to both out- and inpatient hospital contacts, private practicing specialists and other healthcare providers, including diagnostic imaging and cancer diagnostic pathways [1]. Gatekeeping may help manage healthcare costs by reducing unnecessary specialist visits and promoting cost-effective treatments [20]. However, gatekeeping may also have some disadvantages. The secretary is often the first point of contact with general practice, which can result in a double gatekeeper function [21]. Some patients may feel a need to advocate for themselves to get an appointment, further tests or treatments, while others may avoid contact [22].

Perceived access to general practice, the relationships with GPs and their staff, and experiences from previous encounters in general practice may influence healthcare-seeking behaviour [23,24]. Further, these perspectives may differ between groups with different sex, age, morbidity and health literacy. In this study, we hypothesised that females, older individuals and those with existing chronic disease are more likely to prefer being seen by the same doctor and to report previous negative experiences with their GP [18]. Moreover, we hypothesised that individuals with health literacy challenges are more likely to perceive access to general practice as difficult and to have had negative experiences in previous encounters with general practice [25]. Understanding inequities in the perceived access to general practice, relationship with GPs, and previous experiences with general practice may lead to the development of targeted interventions. These interventions can enable differentiated healthcare services, ensuring that resources and support are tailored to the unique circumstances of different groups, thereby reducing inequity. Thus, this study aims to (1) explore perceived access to general practice, relationship with the GP and previous experiences with contact to general practice and (2) analyse the associations between sex, age, chronic disease, health literacy and access to, relationship, and previous experiences with general practice in the Danish population.

## Methods

### Study design and data collection

This study is a cross-sectional study based on the Danish Symptom Cohort II (DaSC II), a large-scale nationwide survey investigating symptom experiences and healthcare-seeking behaviour in the general population [26]. A sample of 100,000 individuals aged 20 years or older was randomly selected using the Danish Civil Registration System (CRS) and invited to participate in an online survey. In the CRS, each individual is given a unique identification number (CRS number) at birth or migration. The invitation was sent to a digital mailbox linked to the CRS number. The invitation letter included a description of the purpose of the study, legal rights and permissions, and a link to the survey. Individuals exempted from digital mail (≈7%) were considered ineligible for the study [27]. Participation was voluntary, and individuals who did not wish to participate had the opportunity to decline online or by contacting the project group. Non-respondents received two reminder letters in their digital mailbox encouraging them to participate after 7 and 14 days, respectively. Data were collected from May to July 2022 [26].

### The questionnaire

The development of the DaSC II questionnaire followed the COnsensus-based Standards for the selection of health Measurement INstruments (COSMIN) guidelines [28]. The questionnaire was pilot tested twice; first in a mixed academic setting with representatives from different disciplines and then by a user panel of twelve representatives from the general population (age range 20–89 years). Prior to the distribution of the final survey, a field test was conducted among 499 randomly selected individuals from the general population. The tests resulted in minor rewordings and shortenings of the questions, and changes in the logistical procedures. The methodological framework, development and details of the entire questionnaire are described elsewhere [26].

The DaSC II questionnaire covered several constructs and consisted of six overall domains investigating different aspects of healthcare-seeking behaviour. These have been described in detail elsewhere [26]. In the present study, we included questions about access to general practice, relationships and previous experiences with GPs. These questions were a part of a domain that explored general aspects of interactions with general practice and GPs, and were not linked to any specific contact or symptom. In the questionnaire development, we made an item bank covering the following three constructs: (1) access to general practice, (2) relationship with the GP and (3) previous experiences with encounters in general practice. The constructs and items were based on existing literature [23,24,29], inspired by the UK Cancer Awareness Measures (CAM) [18] and qualified by interviews with the user panel. The three constructs were selected based on the hypotheses that restricted perceived access, previous negative experience or a poor relationship with the GP may compromise the healthcare-seeking behaviour in the general population, potentially leading to delay or undertreatment. In the final survey, seven statements covering the three constructs were included. Each statement was used as a formative question. Access to general practice comprised three statements: Q1 ‘Difficult to talk to the secretary’, Q2 ‘Difficult getting an appointment’ and Q3 ‘Difficult to get in touch’. The relationship with the GP was covered by two statements: Q4 ‘Confident the doctor can help’ and Q5 ‘Like to be seen by the same doctor’. Finally, experiences with encounters in general practice were covered by: Q6 ‘Negative experiences’ and Q7 ‘Too little time’. The response options to all statements were placed on a four-point rating scale, ranging from ‘Completely agree’ to ‘Completely disagree’. Further, the respondents could choose a ‘not relevant’ option. The wording of all statements appears in Table 1.

**Table 1.** Wording of the statements covering the three constructs access to general practice, relationship with the GP and previous experiences with encounters to general practice.

Access to general practice	
Q1: Difficult to talk to the secretary	I find it difficult to talk to the secretary at the GP’s office about my problems
Q2: Difficulty getting an appointment	I have difficulty getting an appointment with the doctor I want to see
Q3: Difficult to get in touch	I find it difficult to get in touch with my doctor
The relationship with the general practitioner	
Q4: Confident the doctor can help	I am confident that my doctor can help me
Q5: Like to be seen by the same doctor	I would like to be seen by the same doctor I usually see
Previous experiences with encounters in general practice	
Q6: Negative experiences	I have had negative experiences with doctor visits
Q7: Too little time	I often find that the doctor has too little time for my consultation

*Answer categories*: Completely agree, partly agree, partly disagree, completely disagree and not relevant.

### Covariates

We included data about self-reported chronic disease and health literacy from the questionnaire. Chronic disease was determined based on the following question: ‘Do you have any chronic disease, long-term effects after injuries, disability or other chronic disorder?’ Response options were: ‘Yes’, ‘No’ and ‘I don’t know’. Health literacy was evaluated based on the Health Literacy Questionnaire (HLQ) [30,31]. The HLQ is a comprehensive reflective scale comprising 44 items in nine domains, each measuring a dimension of health literacy. The HLQ has been translated, adapted and validated in a Danish context [32]. The following four domains were included in the DaSC II survey: ‘Feel understood and supported by healthcare providers (“Understood and supported”, four items)’, ‘Have sufficient information to manage my health (“Sufficient information”, four items)’, ‘Have social support for health (“Social support”, five items)’ and ‘Ability to actively engage with healthcare providers (“Actively engage”, five items)’. The HLQ items cover a range of health literacy-related statements. For the first three domains, respondents were asked on a four-point scale whether they agreed with each statement (1 = strongly disagree; 2 = disagree; 3 = agree; 4 = strongly agree), and for the last domain they were asked on a five-point Likert scale whether they found the statements difficult or easy (1 = always difficult; 2 = usually difficult; 3 = sometimes difficult; 4 = usually easy; 5 = always easy). Mean score and the appurtenant standard deviation were calculated for each domain. Details of all variables and questions are described in Table S1 in the Supplementary Materials.

Age and sex were obtained from the CRS number at the time of data collection. Socioeconomic data were obtained from Statistics Denmark (Copenhagen, Denmark) by linkage using the CRS-numbers. The variables of interest were marital status, highest obtained level of education, labour market affiliation and ethnicity [33–35]. Individuals with missing data on socioeconomics were assigned to the largest group within each covariate. Data on vital status in the period of data collection were obtained from the Danish Health Data Authority.

### Statistical analyses

Only individuals who had completed all items relevant to the study were included in the analyses. The distribution of answers to each statement was calculated using descriptive statistics. We dichotomised the answers to each statement by merging completely and partly agree/disagree into agree and disagree, respectively. Respondents who had answered ‘not relevant’ were not included in the logistic regression analyses.

We used uni- and multivariable logistic regression models to analyse the associations between the covariates and agreement with each of the seven statements. Covariates considered were sex, age, chronic disease (yes or no) and health literacy. Age was categorised as follows: 20–39 years, 40–59 years, 60–79 years and 80+ years. Respondents who had answered ‘I don’t know’ to the question about chronic disease were allocated to the ‘no’ responses. Health literacy was included as a continuous variable.

The analyses were adjusted in two steps: in model A, we adjusted for sex, age, chronic disease and health literacy. In model B, the analyses were additionally adjusted for the following socioeconomic factors (SES): marital status (single/living alone or married/living together); highest obtained level of education (low (<10 years), middle (10–15 years) or high (≥15 years); labour market affiliation (working, pension, out of workforce, and disability pension) and ethnicity (Danish or immigrants/descendants of immigrants). Only SES that showed significant associations in the crude analyses were included as confounders in model B. The odds ratios (ORs) were calculated with a 95% confidence interval, and *p* values of 0.05 or less were considered statistically significant.

All analyses were conducted in STATA version 18 (StataCorp, College Station, TX).

## Results

### Study population

**Figure 1.**
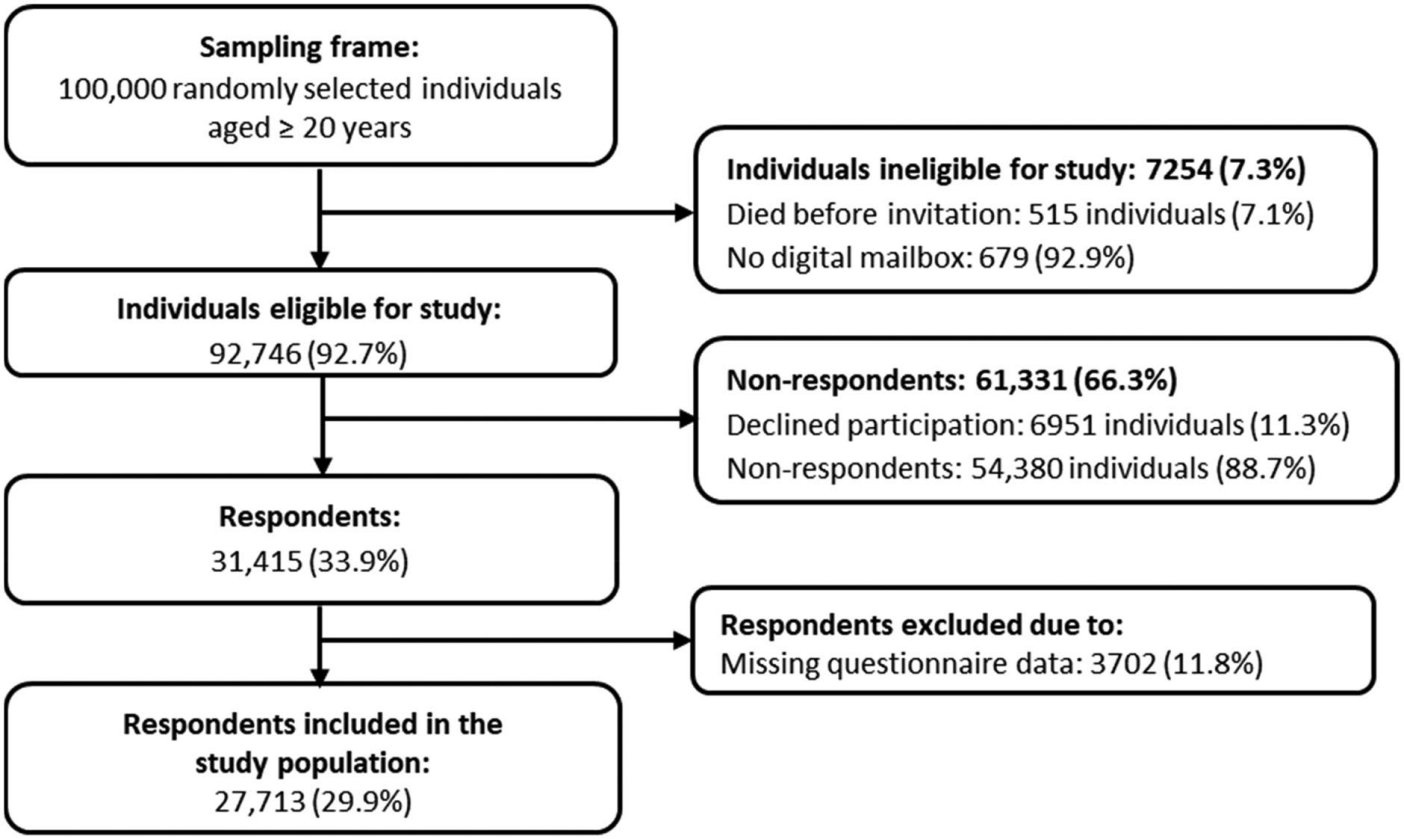
Flowchart of the study population.

Of the 100,000 randomly selected individuals, 7.3% were ineligible due to death before invitation or having no digital mailbox. Of the 92,473 eligible invitees, 31,415 (33.9%) responded. Among those who responded, 27,713 (29.9%) completed all questions of relevance for this study and were included in the study population (Figure 1). The characteristics of the study population are shown in Table 2.

**Table 2.** Sampling frame and study population characteristics.

	Sampling frame		Study population	
	*N* ^a^	%^a^	*N*	%
**Total**	100,000	100.0	27,713	100.0
**Sex**				
Females	50,686	50.7	15,796	57.0
Males	49,314	49.3	11,917	43.0
**Age groups**				
20–39 years	32,720	32.7	5621	20.3
40–59 years	33,474	33.5	10,173	36.7
60–79 years	27,383	27.4	10,748	38.8
80+ years	6423	6.4	1171	4.2
**Chronic disease**				
No	–	–	15,853	57.2
Yes	–	–	11,860	42.8
**Marital status**				
Single/living alone	37,088	37.0	8104	29.2
Married/living together	62,912	63.0	19,609	70.8
**Highest obtained level of education**				
Low (<10 years)	12,032	12.0	1976	7.1
Middle (10–15 years)	52,018	52.0	13,599	49.7
High (>15 years)	35,950	36.0	12,138	43.8
**Labour market affiliation**				
Working	63,202	63.2	17,516	63.2
Pension	22,355	22.4	7298	26.3
Out of workforce	9404	9.4	1873	6.8
Disability pension	5039	5.0	1026	3.7
**Ethnicity**				
Danish	85,329	85.3	25,725	92.8
Immigrants/descendants of immigrants	14,671	14.7	1988	7.2
			Mean sum score	Standard deviation
**Health literacy** ^a^				
‘Understood and supported’	–	–	2.87	0.68
‘Sufficient information’	–	–	3.00	0.58
‘Social support’	–	–	3.05	0.60
‘Actively engage’	–	–	3.74	0.89

^a^ Domains from the Health Literacy Questionnaire.

**Figure 2.**
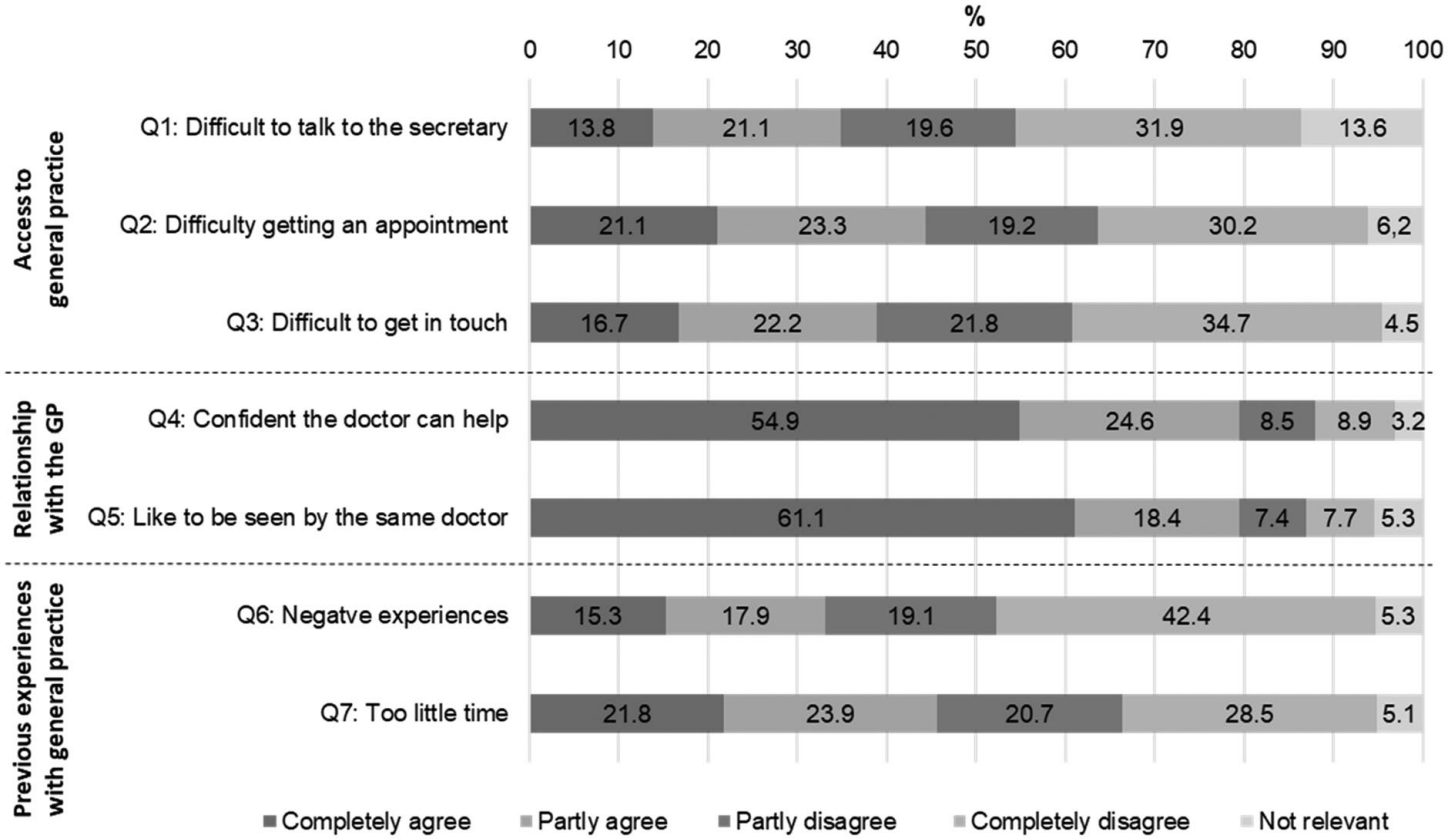
Distribution of answers to the statements regarding access to general practice, relationship with the general practitioner and previous experiences with contact to general practice (*N* = 27,713).

The distribution of answers to each statement is shown in Figure 2. Regarding access to general practice, 34.9% of the respondents completely or partly agreed that they found it ‘difficult to talk to the secretary’ and 44.4% had ‘difficulties getting an appointment’, while 38.9% found it ‘difficult to get in touch’. In total 79.5% were ‘confident that the doctor could help’ and would ‘like to be seen by the same doctor’. At total of 33.2% reported previous ‘negative experiences’, 45.7% agreed to experiencing ‘too little time’ (Figure 2).

Tables 3 and 4 show the associations between sex, age, chronic disease, the four health literacy domains, and each of the seven statements based on both model A and model B. Corresponding results from the crude analyses are reported in Tables S2 and S3 in the Supplementary Material. The ORs presented in the following are all based on model B. No significant differences were found between the models.

**Table 3.** Adjusted associations between sex, age, chronic disease and each of the seven statements regarding access, relationship and previous experiences with encounters in general practice.

	Access to general practice											
	*Q1*: Difficult to talk to the secretary (*n* = 23,944)			*Q2*: Difficulty getting an appointment (*n* = 25,995)			*Q3*: Difficult to get in touch (*n* = 26,466)					
	Agree, *n* (%)	Adj. OR^a^ (95% CI)	Adj. OR^b^ (95% CI)	Agree, *n* (%)	Adj. OR^a^ (95% CI)	Adj. OR^b^ (95% CI)	Agree, *n* (%)	Adj. OR^a^ (95% CI)	Adj. OR^b^ (95% CI)			
**Total**	9556 (40.2)			12,295 (47.3)			10,777 (40.7)					
**Sex**												
Females	5996 (43.4)	Ref.	Ref.	7608 (51.1)	Ref.	Ref.	10777 (40.7)	Ref.	Ref.			
Males	3660 (36.1)	**0.79 (0.75–0.84)**	**0.78 (0.74–0.83)**	4687 (42.2)	**0.75 (0.71–0.79)**	**0.73 (0.69–0.77)**	6672 (44.0)	**0.77 (0.73–0.82)**	**0.75 (0.71–0.80)**			
**Age groups**												
20–39 years	2238 (44.2)	Ref.	Ref.	2611 (50.1)	Ref.	Ref.	2250 (42.1)	Ref.	Ref.			
40–59 years	3758 (41.8)	1.06 (0.99–1.15)	1.05 (0.97–1.14)	5089 (53.2)	**1.33 (1.24–1.43)**	**1.26 (1.17–1.36)**	4478 (46.0)	**1.42 (1.32–1.53)**	**1.32 (1.22–1.42)**			
60–79 years	3355 (37.6)	1.07 (0.99–1.15)	**1.10 (1.00–1.22)**	4238 (41.8)	0.98 (0.91–1.06)	1.05 (0.96–1.16)	3722 (36.2)	**1.13 (1.05–1.21)**	**1.14 (1.04–1.25)**			
80+ years	305 (31.6)	0.86 (0.74–1.01)	0.92 (0.77–1.11)	357 (32.8)	**0.70 (0.61–0.81)**	**0.83 (0.70–0.98)**	327 (29.6)	0.89 (0.77–1.03)	0.98 (0.82–1.17)			
**Chronic disease**												
No	5386 (39.7)	Ref.	Ref.	6872 (46.8)	Ref.	Ref.	6082 (40.6)	Ref.	Ref.			
Yes	4270 (41.1)	0.97 (0.92–1.03)	0.98 (0.92–1.04)	5423 (48.0)	**1.06 (1.00–1.11**)	**1.09 (1.03–1.15)**	4695 (41.0)	1.01 (0.95–1.06)	1.04 (0.98–1.10)			
	Relationship with the general practitioner						Previous experiences with encounters in general practice					
	*Q4*: Confident the doctor can help (*n* = 26,826)			*Q5*: Like to be seen by the same doctor (*n* = 26,244)			*Q6*: Negative experiences (*n* = 26,244)			*Q7*: Too little time (*n* = 26,300)		
	Agree, *n* (%)	Adj. OR^a^ (95% CI)	Adj. OR^b^ (95% CI)	Agree, *n* (%)	Adj. OR^a^ (95% CI)	Adj. OR^b^ (95% CI)	Agree, *n* (%)	Adj. OR^a^ (95% CI)	Adj. OR^b^ (95% CI)	Agree, *n* (%)	Adj. OR^a^ (95% CI)	Adj. OR^b^ (95% CI)
**Total**	22,027 (82.1)			22,043 (84.0)			9190 (35.0)			12,674 (48.2)		
**Sex**												
Females	12550 (81.8)	Ref.	Ref.	12865 (85.6)	Ref.	Ref.	6013 (40.1)	Ref.	Ref.	7941 (52.7)	Ref.	Ref.
Males	9477 (82.4)	1.01 (0.94–1.08)	1.00 (0.93–1.06)	9178 (81.9)	**0.75 (0.70–0.80)**	**0.75 (0.70–0.80)**	3177 (28.2)	**0.64 (0.60–0.68)**	**0.65 (0.61–0.69)**	4733 (42.1)	**0.69 (0.66–0.73)**	**0.69 (0.65–0.73)**
**Age groups**												
20–39 years	4366 (81.3)	Ref.	Ref.	4148 (80.2)	Ref.	Ref.	2429 (45.4)	Ref.	Ref.	2634 (50.0)	Ref.	Ref.
40–59 years	8175 (83.0)	1.01 (0.92–1.10)	0.98 (0.89–1.08)	8154 (84.8)	**1.40 (1.28–1.54)**	**1.37 (1.24–1.50)**	3693 (38.0)	**0.82 (0.76–0.88)**	**0.82 (0.75–0.88)**	5085 (52.4)	**1.34 (1.25–1.44)**	**1.30 (1.20–1.41)**
60–79 years	8604 (82.2)	**0.81 (0.74–0.88)**	**0.86 (0.76–0.97)**	8806 (85.2)	**1.46 (1.33–1.60)**	**1.39 (1.23–1.56)**	2839 (28.1)	**0.61 (0.56–0.66)**	**0.64 (0.58–0.70)**	4556 (44.5)	**1.17 (1.08–1.26)**	**1.26 (1.14–1.38)**
80+ years	882 (77.8)	**0.57 (0.49–0.67)**	**0.66 (0.54–0.80)**	935 (83.5)	**1.30 (1.09–1.55)**	**1.24 (1.01–1.53)**	229 (21.6)	**0.46 (0.38–0.54)**	**0.50 (0.41–0.61)**	399 (36.3)	0.87 (0.75–1.01)	1.03 (0.87–1.22)
**Chronic disease**												
No	12700 (83.2)	Ref.	Ref.	12360 (83.0)	Ref.	Ref.	4692 (31.4)	Ref.	Ref.	6746 (45.3)	Ref.	Ref.
Yes	9327 (80.7)	0.87 (0.81–0.93)	**0.88 (0.82–0.94)**	9683 (85.4)	**1.11 (1.03–1.19)**	**1.11 (1.03–1.19)**	4498 (39.8)	**1.55 (1.46–1.64)**	**1.50 (1.41–1.59)**	5928 (51.9)	**1.27 (1.20–1.34)**	**1.28 (1.21–1.36)**

Bold statistically significant, *p* value <0.05.

^a^ *Model A*: Adjusted (Adj.) for sex, age, chronic disease and each Health Literacy Questionnaire domain (health literacy)

^b^ *Model B*: Adjusted for sex, age, chronic disease and health literacy and socioeconomics in terms of marital status, highest obtained educational level, labour market affiliation and ethnicity

**Table 4.** Adjusted associations between health literacy and each of the seven statements regarding access, relationship and previous experiences with encounters in general practice

	Access to general practice							
	*Q1*: Difficult to talk to the secretary (*n* = 23,944)		*Q2*: Difficulty getting an appointment (*n* = 25,995)		*Q3*: Difficult to get in touch (*n* = 26,466)			
	Adj. OR^a^ (95% CI)	Adj. OR^b^ (95% CI)	Adj. OR^a^ (95% CI)	Adj. OR^b^ (95% CI)	Adj. OR^a^ (95% CI)	Adj. OR^b^ (95% CI)		
**Health literacy**								
‘Understood and supported’	0.99 (0.94–1.06)	0.99 (0.93–1.05)	**0.64 (0.61–0.68)**	**0.66 (0.62–0.69)**	**0.59 (0.56–0.62)**	**0.60 (0.57–0.63)**		
‘Sufficient information’	1.01 (0.94–1.08)	1.02 (0.95–1.09)	1.05 (0.98–1.12)	1.03 (0.96–1.09)	**1.09 (1.02–1.17)**	**1.08 (1.01–1.15)**		
‘Social support’	**0.91 (0.85–0.97)**	**0.91 (0.85–0.97)**	**1.14 (1.08–1.22)**	**1.10 (1.03–1.17)**	**1.18 (1.11–1.25)**	**1.13 (1.06–1.20)**		
‘Actively engage’	**0.44 (0.42–0.46)**	**0.44 (0.42–0.46)**	**0.58 (0.55–0.60)**	**0.57 (0.55–0.59)**	**0.55 (0.53–0.57)**	**0.54 (0.52–0.56)**		
	Relationship with the general practitioner				Previous experiences with encounters in general practice			
	*Q4*: Confident the doctor can help (*n* = 26,826)		*Q5*: Like to be seen by the same doctor (*n* = 26,244)		*Q6*: Negative experiences (*n* = 26,244)		*Q7*: Too little time (*n* = 26,300)	
	Adj. OR^a^ (95% CI)	Adj. OR^b^ (95% CI)	Adj. OR^a^ (95% CI)	Adj. OR^b^ (95% CI)	Adj. OR^a^ (95% CI)	Adj. OR^b^ (95% CI)	Adj. OR^a^ (95% CI)	Adj. OR^b^ (95% CI)
**Health literacy**								
‘Understood and supported’	**1.59 (1.49–1.70)**	**1.62 (1.51–1.73)**	**1.32 (1.23–1.41)**	**1.32 (1.23–1.42)**	**0.76 (0.71–0.80)**	**0.76 (0.71–0.80)**	**0.76 (0.72–0.81)**	**0.77 (0.72–0.81)**
‘Sufficient information’	1.03 (0.95–1.11)	1.02 (0.94–1.10)	**0.90 (0.83–0.97)**	**0.89 (0.82–0.97)**	**1.13 (1.06–1.21)**	**1.11 (1.04–1.19)**	**1.08 (1.01–1.15)**	**1.07 (1.00–1.15)**
‘Social support’	**0.92 (0.85–0.98)**	**0.89 (0.83–0.95)**	**0.94 (0.87–1.02)**	**0.89 (0.83–0.95)**	**1.07 (1.00–1.13)**	**1.07 (1.00–1.14)**	**1.07 (1.00–1.13)**	**1.06 (1.00–1.13)**
‘Actively engage’	**1.48 (1.41–1.55)**	**1.47 (1.40–1.54)**	**0.87 (0.83–0.91)**	**0.86 (0.82–0.91)**	**0.42 (0.41–0.44)**	**0.42 (0.41–0.44)**	**0.43 (0.41–0.45)**	**0.43 (0.41–0.45)**

Bold statistically significant, *p* value <0.05.

^a^ *Model A*: Adjusted (Adj.) for sex, age, chronic disease and each Health Literacy Questionnaire domain (health literacy).

^b^ *Model B*: Adjusted for sex, age, chronic disease and health literacy and socioeconomics in terms of marital status, highest obtained educational level, labour market affiliation and ethnicity.

### Access to general practice

Males had lower odds of reporting ‘difficulties talking to the secretary’ (OR 0.78, 95% CI: 0.74–0.83), ‘difficulties getting an appointment’ (OR 0.73, 95% CI: 0.69–0.77) and ‘difficult to get in touch’ (OR 0.75, 95% CI: 0.71–0.80) compared to females. Individuals with a chronic disease had higher odds of agreeing with ‘difficulties getting an appointment’ (OR 1.09, 95% CI: 1.03–1.15) compared to individuals with no chronic disease (Table 3).

Related to health literacy, a higher score for being able to ‘actively engage’ with healthcare providers was associated with lower odds of reporting difficulties with access to general practice, whereas individuals with higher score for having ‘social support’ for health had higher odds of reporting ‘difficulties with getting an appointment’ (OR 1.10. 95% CI: 1.03–1.17) and ‘difficulties getting in touch’ with the doctor (OR 1.13. 95% CI: 1.06–1.20), Table 3.

### The relationship with the general practitioner

Males had lower odds of would ‘like to be seen by the same doctor’ (OR 0.75, 95% CI: 0.70–0.80) than females, while individuals with older age (OR_80+_ 1.24, 95% CI: 1.01–1.53) and a chronic disease (OR 1.11, 95% CI: 1.03–1.19) had higher odds. The likelihood of being ‘confident the doctor can help’ was lower among individuals with higher age (OR_80+_ 0.66, 95% CI: 0.54–0.80) and individuals with a chronic disease (OR 0.88, 95% CI: 0.82–0.94), Table 3.

Related to health literacy, a higher score for feeling ‘understood and supported’ by healthcare providers increased the likelihood of being ‘confident the doctor can help’ (OR 1.62, 95% CI: 1.51–1.73) and ‘like to be seen by the same doctor’ (OR 1.32, 95% CI: 1.23–1.42). Being able to ‘actively engage’ increased the odds of being ‘confident the doctor can help’ (OR 1.47, 95% CI: 1.40–1.54) and lowered the odds of ‘like to be seen by the same doctor’ (OR 0.86, 95% CI: 0.82–0.91). Individuals with higher score for ‘social support’ had lower odds of being ‘confident the doctor can help’ (OR 0.89, 95% CI: 0.83–0.95), and higher score for having ‘sufficient information’ lowered the odds of ‘like to be seen by the same doctor’ (OR 0.89, 95% CI 0.82–0.97), Table 4.

### Previous experiences with contact to general practice

Males were less likely to report previous ‘negative experiences’ (OR 0.65, 95% CI: 0.61–0.69) and ‘too little time’ (OR 0.73, 95% CI: 0.69–0.77). Older age decreased the likelihood of reporting previous ‘negative experiences’ and increased the likelihood of experiencing ‘too little time’, whereas having a chronic disease increased the likelihood of agreeing with both (Table 3).

Related to health literacy, higher scores for feeling ‘understood and supported’ decreased the likelihood of reporting previous ‘negative experiences’ (OR 0.76, 95% CI: 0.71–0.80) and reporting ‘too little time’ (OR 0.77, 95% CI: 0.77–0.81). Likewise, higher score for being able to ‘actively engage’ was associated with lower odds of reporting previous ‘negative experiences’ (OR 0.42, 95% CI: 0.41–0.44) and ‘too little time’ (OR 0.43, 95% CI: 0.41–0.45) (Table 4).

## Discussion

### Main findings

In the present study, we explored perceived access to general practice, the relationship with the GP, and experiences from previous encounters in general practice. One out of three respondents reported difficulties with perceived access to general practice, whereas eight out of ten were confident their doctor could help them and preferred to be seen by the same doctor. Notably, one-third reported negative experiences with previous GP visits, and nearly half reported that the doctor had insufficient time for the consultations.

Females were more likely to report difficulties with access and previous negative experiences with contact to general practice. Individuals with higher age and those who reported existing chronic disease(s) were less likely to be confident that the doctor could help.

Individuals with health literacy challenges, in terms of being less able to actively engage with healthcare providers, were more likely to report difficulties with access and negative experiences with previous contacts to general practice. They were also less likely to be confident that the doctor could help them. The same applied for individuals who reported feeling less understood by healthcare providers. Individuals reporting high ability to actively engage and sufficient information to manage their own health were more likely to desire being seen by the same doctor.

## Discussion of results and comparison with existing literature

In the present study, the proportion of individuals reporting difficulties with access to general practice seemed slightly lower than in a UK study based on the CAM [29]. Even though the questions in the present study were inspired by the CAM, the wordings and settings of the two studies differ, thus hampering direct comparison of estimates.

Females were more likely to report challenges with perceived access, preferring to be seen by the same GP and to have had previous negative experiences than males. This is similar to the findings by Moffat et al. [18] and may be explained by differences in symptom perceptions, healthcare-seeking behaviour, preferences and expectations between the sexes [26,36]. Females report more symptoms than males and have a higher absolute number of contacts with the healthcare system [26]. This increases their risk of negative experiences, and though counterintuitive, it may also become a barrier for some, because individuals strive to be good citizens by appropriately using healthcare resources [29,37].

Individuals with existing chronic disease were more likely to have had negative experiences with doctor visits. This may imply, that their needs are not always met, as described by Schwarz et al. [15], but may also partly be explained by their number of contacts with the healthcare system being higher, thus enhancing the risk of a previous negative experience. We do not know the number of negative experiences the respondents have had, nor whether they have had just as many or more positive experiences. Schwarz et al. suggest that limited consultation time, health literacy challenges, fear of stigmatisation and previous negative experiences are of importance, and, often invisible, barriers to healthcare seeking among individuals with chronic disease [15]. These barriers may influence both treatment and follow-up, thereby affecting the quality of care and quality of life [38]. Individuals with health literacy challenges, such as feeling less understood and supported by healthcare providers were more likely to report difficulties with access to general practice and previous negative experiences. Further, they were less likely to be confident that the GP could help them. This is in line with previous studies on cancer diagnostics [39], healthcare-seeking behaviour in the general population [40] and chronic care in general practice [41], emphasising that health literacy significantly impacts healthcare access and outcomes, making it a crucial factor in addressing health inequities.

In the present study, individuals who reported that they felt understood and supported by healthcare providers were more than 50% more likely to be confident that their GP could help them. This indicates that the relationships between the GP, the staff and the patients are of substantial significance for the overall collaboration and trust. Studies have shown that health literacy challenges are associated with risk behaviours and socioeconomic deprivation [3,7]. Individuals in these groups may experience stigmatisation when consulting general practice, which compromise the relationship between the patients and the healthcare professionals. This, in turn, increases the risk of these individuals avoiding or postponing healthcare seeking, even when necessary [42]. In line with existing literature, this study emphasises that the ability to actively engage with healthcare professionals is a key element for succeeding as a patient in the healthcare system [43,44]. The capability to engage entails verbalising symptoms and health challenges, as well as the comprehension of information and questions from healthcare professionals. Strengthening this domain of health literacy must be emphasised in future initiatives targeting inequity in both health and healthcare-seeking behaviour encompassing that one size does not fit all. Even though this may seem to be an individual responsibility, it must be underlined that this is not the case. Strengthening organisational health literacy is just as important, and to a high degree a task for communities and the healthcare system [45,46]. Organisational health literacy is defined as ‘the way in which services, organizations and systems make health information and resources available and accessible to people according to health literacy strengths and limitations’ [47]. From a primary care perspective one way to improve organisational health literacy responsiveness could be for GPs and their staff to be proactive in establishing or maintaining contact with certain patients. This is a time-consuming task, necessitating the de-prioritisation or delegating of other responsibilities.

In the present study, older age was associated with lower confidence in the GP’s ability to help. This may be due to several reasons. Some older individuals may have realised that not all symptoms or diseases can be cured, while others may view symptoms and deteriorating health a natural consequence of aging, beyond the doctor’s ability to address [37].

## Strengths and limitations of the study

The large sample size of 100,000 individuals randomly selected from the general population is considered a strength. The response rate was lower than desired, but comparable to another large-scale study conducted during the same period. The respondents were in general more likely to be female, middle aged and to have a higher educational level and Danish ethnicity compared to non-respondents. Yet, the respondents included representatives of all ages and socioeconomic backgrounds, enabling the analysis of subgroups [26]. Nevertheless, selection bias must be considered. Individuals with high knowledge about health or strong opinions about the healthcare system may be more likely to answer a questionnaire about symptoms and healthcare-seeking behaviour [48]. If individuals with previous negative experiences or a strained relationship with the healthcare system are more likely to respond, or if negative experiences are easier to recall, we might have overestimated the challenges related to access and previous negative experiences. Yet, the high proportion of respondents who reported a good relationship with their GP does not support this assumption. Other barriers towards contact to general practice also exist and could have been relevant to explore. Thus, another limitation of this study is that it only addresses some of the possible barriers.

Health literacy challenges have been associated with lower research participation rates [49]. However, the mean sum scores for the HLQ domains in the DaSC II study were similar to the scores in the validation study by Maindal et al. where data were collected through face-to-face interviews [31,32]. Non-respondents and individuals’ ineligible for study, such as those exempted from digital mail, may experience more health literacy challenges. This could lead to an underestimation of the inequities in perceived access to general practice, and previous negative experiences with encounters in general practice in the present study. In the field test, individuals exempted from digital mail were invited by postal letter. However, very few were able to respond to the questionnaire. Additionally, the project group was contacted by several relatives of the postal invitees requesting their exclusion from study. Thus, the postal invitation was omitted in the final distribution to avoid unnecessary disturbance. Consequently the older individuals among the respondents may be in better health than the non-respondents of older age inducing a possible selection bias [48]. The response rate was sought heightened by a thorough questionnaire development process following the COSMIN guidelines ensuring relevance and feasibility, two pilot tests ensuring high face and content validity, an easily read and understandable invitation and the possibility of contacting the project group throughout the data collection period, and by drawing lots for gift certificates among the respondents [26].

The three constructs included as outcomes in this study, included statements related to both the GP, secretary and to general practice as an organisation including both GPs and staff members. This distinction is important, since the organisation of general practice has changed, and many consultations are now conducted by other than the doctors. For instance, chronic care consultations are often partly managed by practice staff in Denmark. During the development of the questionnaire, we operationalised the three constructs into: access to general practice, the relationship with the GP, and previous experiences with encounters in general practice. Although the individual statements related to previous experiences focused on the doctor and doctor visits, we considered this as related to both the GPs and the other health professionals in general practice. In the pilot tests, previous experiences with the GP were understood as encompassing both previous experiences with the doctor and the staff in general practice. Thus, we considered naming the construct ‘…in general practice’ reasonable. However, we cannot exclude the possibility that patients with chronic diseases were more likely to report difficulties in getting an appointment with their preferred doctor due to chronic care consultations being carried out by practice staff.

The HLQ is widely distributed all over the world making it possible to compare findings with existing literature and discuss health literacy challenges across different populations [50]. The HLQ does not provide a total score or cut-off for high or low health literacy, thus allowing the same individual to possess both health literacy strengths in some domains and challenges with other domains [51]. All four HLQ domains included in the present study showed good internal consistency in the validation [32]. It could be argued that some of the statements reflecting the three constructs, such as difficulties with getting an appointment, also pertain to aspects of health literacy. Nevertheless, individuals may possess both strengths and limitations in health literacy. Therefore, we found it reasonable to examine the associations between health literacy, as measured by the HLQ domains, and the constructs included in the present study.

We used self-reported chronic disease as a proxy for morbidity. Chronic disease can be measured in several ways [52–54], and some participants who did not feel ill may have answered ‘no’ to the question even though they have a disease registered in the national registers. Although it can induce some misclassification, self-perceived chronic disease probably has a greater influence on the individual’s need for contacting the GP and the related perception of access to the healthcare system than register-based morbidity indexes [55]. We considered determining the presence of chronic disease by obtaining data from registers. However, this method also has limitations. For instance, the national registers do not contain information about diagnoses for patients who are diagnosed, treated and monitored only in general practice, thus register-based comorbidity indexes can also lead to misclassification [56].

The present study was an explorative epidemiological study including multiple comparisons. Thus, some risk of type I error due to multiple testing cannot be excluded.

## Implications

Healthcare-seeking behaviour and access to the healthcare system is not uniformly equitable. Hence, a focus on individuals or groups facing challenges in navigating in the organisation of the healthcare system, that is when initiating contact to general practice, could be beneficial in terms of reducing inequity.

To strengthen health literacy and promote equity in access to healthcare services, it is essential to allocate sufficient time and provide guidance to those in need. This can be supported, for instance, by enhancing organisational health literacy responsiveness within primary care settings. For example, GPs and staff in general practice are often well acquainted with their patients giving them the opportunity to prioritise the allocation of time and efforts to those who need them. Making small changes in the everyday structure to reinforce the possibilities of individualisation for example, by taking organisational health literacy into account, could be valuable in reducing inequity, regardless of how the GP clinic is otherwise organised. Initiatives supporting differentiation of patients have been suggested in the recent report from the Danish Health Structure Commission [57], framing that a political focus on supporting structural changes and allocating the necessary resources is a paramount requisite to improve equity in health.

The challenges with access to general practice found in the present study, indicate that both the mutual expectations, the communication and the organisation of each clinic may be crucial in facilitating adequate healthcare-seeking behaviour. In recent years, various new methods of organising general practice have emerged, including online consultations, team-based care and task delegations. Solutions like telemedicine may be beneficial for some patients but challenging for others. Differentiation between patients and consultation types is necessary and aligns with both political and scientific trends.

## Conclusions

This study highlighted difficulties with access to general practice and previous negative experiences with GP contact. However, it also revealed that the Danish population has high confidence in their GP. Females, individuals with chronic disease and individuals with health literacy challenges, particular in terms of being less able to actively engage with healthcare providers, were more likely to report difficulties with perceived access to and previous negative experiences with encounters in general practice. This underscores the importance of strengthening both individual and organisational health literacy. The study also emphasised the importance of the relationship between GPs and patients and the continuity of care provider in general practice. This was particularly significant for individuals with a chronic disease and health literacy challenges, suggesting that equity in health may be compromised by inequities in perceived access to primary care.

## Supplementary Material

SUPPLEMENTARY MATERIAL.docx

## Data Availability

The datasets generated and analysed in the current study are not publicly available and cannot be shared due to the data protection regulations of the Danish Data Protection Agency. Access to data is strictly limited to the researchers, who have obtained permission for data processing. This permission was given to the Department of Public Health, University of Southern Denmark. Further enquiries can be made to PI Dorte Jarbøl, email: DJarbol@health.sdu.dk.

## References

[1] Pedersen KM, Andersen JS, Søndergaard J. General practice and primary health care in Denmark. J Am Board Fam Med. 2012;25(Suppl. 1):S34–S38. doi:10.3122/jabfm.2012.02.110216. 22403249

[2] Balasubramaniam K, Rasmussen S, Haastrup PF, et al. Dealing with symptoms in the general population: lessons learned from the Danish Symptom Cohort. Br J Gen Pract. 2022;72(723):460–461. doi:10.3399/bjgp22X720713.

[3] Pedersen SE, Aaby A, Friis K, et al. Multimorbidity and health literacy: a population-based survey among 28,627 Danish adults. Scand J Public Health. 2021;51(2):165–172. 34636270 10.1177/14034948211045921

[4] Aaby A, Beauchamp A, O’Hara J, et al. Large diversity in Danish health literacy profiles: perspectives for care of long-term illness and multimorbidity. Eur J Public Health. 2020;30(1):75–80. doi:10.1093/eurpub/ckz134. 31363738

[5] Brach C, Keller D, Hernandez LM, et al. Ten attributes of health literate health care organizations. Washington (DC): National Academy of Medicine; 2012.

[6] Svendsen MT, Bak CK, Sørensen K, et al. Associations of health literacy with socioeconomic position, health risk behavior, and health status: a large national population-based survey among Danish adults. BMC Public Health. 2020;20(1):565. doi:10.1186/s12889-020-08498-8. 32345275 PMC7187482

[7] Paasche-Orlow MK, Wolf MS. The causal pathways linking health literacy to health outcomes. Am J Health Behav. 2007;31(Suppl. 1):S19–S26. doi:10.5993/AJHB.31.s1.4. 17931132

[8] Protheroe J, Whittle R, Bartlam B, et al. Health literacy, associated lifestyle and demographic factors in adult population of an English city: a cross-sectional survey. Health Expect. 2017;20(1):112–119. doi:10.1111/hex.12440. 26774107 PMC5217902

[9] Mitchell SE, Sadikova E, Jack BW, et al. Health literacy and 30-day postdischarge hospital utilization. J Health Commun. 2012;17(Suppl. 3):325–338. doi:10.1080/10810730.2012.715233. 23030580

[10] Humphrys E, Burt J, Rubin G, et al. The influence of health literacy on the timely diagnosis of symptomatic cancer: a systematic review. Eur J Cancer Care. 2019;28(1):e12920. doi:10.1111/ecc.12920. PMC6559266 30324636

[11] Samoil D, Kim J, Fox C, et al. The importance of health literacy on clinical cancer outcomes: a scoping review. Ann Cancer Epidemiol. 2021;5:3. doi:10.21037/ace-20-30.

[12] Friis K, Aaby A, Lasgaard M, et al. Low health literacy and mortality in individuals with cardiovascular disease, chronic obstructive pulmonary disease, diabetes, and mental illness: a 6-year population-based follow-up study. Int J Environ Res Public Health. 2020;17(24):9399. doi:10.3390/ijerph17249399. 33333909 PMC7765354

[13] Whitaker KL, Scott SE, Wardle J. Applying symptom appraisal models to understand sociodemographic differences in responses to possible cancer symptoms: a research agenda. Br J Cancer. 2015;112(Suppl. 1):S27–S34. doi:10.1038/bjc.2015.39. 25734385 PMC4385973

[14] Sætre LMS, Rasmussen S, Balasubramaniam K, et al. A population-based study on social inequality and barriers to healthcare-seeking with lung cancer symptoms. NPJ Prim Care Respir Med. 2022;32(1):48. doi:10.1038/s41533-022-00314-7. 36335123 PMC9637082

[15] Schwarz T, Schmidt AE, Bobek J, et al. Barriers to accessing health care for people with chronic conditions: a qualitative interview study. BMC Health Serv Res. 2022;22(1):1037. doi:10.1186/s12913-022-08426-z. 35964086 PMC9375930

[16] Elnegaard S, Pedersen AF, Sand Andersen R, et al. What triggers healthcare-seeking behaviour when experiencing a symptom? Results from a population-based survey. BJGP Open. 2017;1(2):bjgpopen17X100761. doi:10.3399/bjgpopen17X100761. PMC6169954 30564656

[17] Hay MC. Reading sensations: understanding the process of distinguishing ‘fine’ from ‘sick’. Transcult Psychiatry. 2008;45(2):198–229. doi:10.1177/1363461508089765. 18562493

[18] Moffat J, Hinchliffe R, Ironmonger L, et al. Identifying anticipated barriers to help-seeking to promote earlier diagnosis of cancer in Great Britain. Public Health. 2016;141:120–125. doi:10.1016/j.puhe.2016.08.012. 27931986 PMC5157686

[19] Ammitzbøll G, Levinsen AKG, Kjær TK, et al. Socioeconomic inequality in cancer in the Nordic countries. A systematic review. Acta Oncol. 2022;61(11):1317–1331. doi:10.1080/0284186X.2022.2143278. 36369792

[20] Sripa P, Hayhoe B, Garg P, et al. Impact of GP gatekeeping on quality of care, and health outcomes, use, and expenditure: a systematic review. Br J Gen Pract. 2019;69(682):e294–e303. doi:10.3399/bjgp19X702209. 30910875 PMC6478478

[21] Ward J, McMurray R. The unspoken work of general practitioner receptionists: a re-examination of emotion management in primary care. Soc Sci Med. 2011;72(10):1583–1587. doi:10.1016/j.socscimed.2011.03.019. 21501912

[22] Churchill R, Allen J, Denman S, et al. Do the attitudes and beliefs of young teenagers towards general practice influence actual consultation behaviour? Br J Gen Pract. 2000;50(461):953–957. 11224965 PMC1313880

[23] Power E, Connor K, Crawford C. Cancer awareness measure 2017. United Kingdom: Cancer Intelligence Team; 2017.

[24] McCutchan GM, Wood F, Edwards A, et al. Influences of cancer symptom knowledge, beliefs and barriers on cancer symptom presentation in relation to socioeconomic deprivation: a systematic review. BMC Cancer. 2015;15(1):1000. doi:10.1186/s12885-015-1972-8. 26698112 PMC4688960

[25] Hedelund Lausen L, Smith SK, Cai A, et al. How is health literacy addressed in primary care? Strategies that general practitioners use to support patients. J Commun Healthc. 2018;11(4):278–287. doi:10.1080/17538068.2018.1531477.

[26] Sætre LMS, Raasthøj I, Lauridsen GB, et al. Revisiting the symptom iceberg based on the Danish symptom cohort – symptom experiences and healthcare-seeking behaviour in the general Danish population in 2022. Heliyon. 2024;10(10):e31090. doi:10.1016/j.heliyon.2024.e31090. 38803940 PMC11128908

[27] Agency for Digital Government. Fritagelse for digital post (excemption from Digital Mail); 2023. Available from: https://www.borger.dk/internet-og-sikkerhed/digital-post/fritagelse-fra-digital-post

[28] Mokkink LB, Terwee CB, Patrick DL, et al. The COSMIN study reached international consensus on taxonomy, terminology, and definitions of measurement properties for health-related patient-reported outcomes. J Clin Epidemiol. 2010;63(7):737–745. doi:10.1016/j.jclinepi.2010.02.006. 20494804

[29] Connor K, Hudson B, Power E. Awareness of the signs, symptoms, and risk factors of cancer and the barriers to seeking help in the UK: comparison of survey data collected online and face-to-face. JMIR Cancer. 2020;6(1):e14539. doi:10.2196/14539. 31951219 PMC6996748

[30] Osborne RH, Batterham RW, Elsworth GR, et al. The grounded psychometric development and initial validation of the Health Literacy Questionnaire (HLQ). BMC Public Health. 2013;13(1):658. doi:10.1186/1471-2458-13-658. 23855504 PMC3718659

[31] Sætre LMS, Jarbøl D, Raasthøj IP, et al. Examining health literacy in the Danish general population: a cross-sectional study on the associations between individual factors and healthcare-seeking behaviour. Eur J Public Health. 2024;34(6):1125–1133. doi:10.1093/eurpub/ckae150. 39402975 PMC11631385

[32] Maindal HT, Kayser L, Norgaard O, et al. Cultural adaptation and validation of the Health Literacy Questionnaire (HLQ): robust nine-dimension Danish language confirmatory factor model. Springerplus. 2016;5(1):1232. doi:10.1186/s40064-016-2887-9. 27536516 PMC4971008

[33] Jensen VM, Rasmussen AW. Danish education registers. Scand J Public Health. 2011;39(7 Suppl.):91–94. doi:10.1177/1403494810394715. 21775362

[34] Pedersen CB. The Danish Civil Registration System. Scand J Public Health. 2011;39(7 Suppl.):22–25. doi:10.1177/1403494810387965. 21775345

[35] Petersson F, Baadsgaard M, Thygesen LC. Danish registers on personal labour market affiliation. Scand J Public Health. 2011;39(7 Suppl.):95–98. doi:10.1177/1403494811408483. 21775363

[36] Ballering AV, Olde Hartman TC, Verheij R, et al. Sex and gender differences in primary care help-seeking for common somatic symptoms: a longitudinal study. Scand J Prim Health Care. 2023;41(2):132–139. doi:10.1080/02813432.2023.2191653. 36995265 PMC10193899

[37] Offersen SMH, Vedsted P, Andersen RS. ‘The Good Citizen’: balancing moral possibilities in everyday life between sensation, symptom and healthcare seeking. Anthropol Act. 2017;24(1):6–12. doi:10.3167/aia.2017.240102.

[38] Olaisen RH, Schluchter MD, Flocke SA, et al. Assessing the longitudinal impact of physician–patient relationship on functional health. Ann Fam Med. 2020;18(5):422–429. doi:10.1370/afm.2554. 32928758 PMC7489969

[39] Petersen GS, Laursen SGW, Jensen H, et al. Patients’ health literacy is associated with timely diagnosis of cancer—a cross-sectional study in Denmark. Eur J Cancer Care. 2022;31(1):e13532. doi:10.1111/ecc.13532. 34704640

[40] Sele Sætre LM, Balasubramaniam K, Aaby A, et al. Health literacy and healthcare seeking with lung cancer symptoms among individuals with different smoking statuses: a population-based study. Eur J Cancer Care. 2024;2024(1):7919967. doi:10.1155/2024/7919967.

[41] Paust A, Lau SR, Bro F, et al. Temporal capital and unaligned times as inequality mechanisms: a case study of chronic care in general practice. Soc Sci Med. 2023;338:116337. doi:10.1016/j.socscimed.2023.116337. 37918228

[42] Nyblade L, Stockton MA, Giger K, et al. Stigma in health facilities: why it matters and how we can change it. BMC Med. 2019;17(1):25. doi:10.1186/s12916-019-1256-2. 30764806 PMC6376713

[43] Friis K, Lasgaard M, Osborne RH, et al. Gaps in understanding health and engagement with healthcare providers across common long-term conditions: a population survey of health literacy in 29,473 Danish citizens. BMJ Open. 2016;6(1):e009627. doi:10.1136/bmjopen-2015-009627. PMC4735217 26769783

[44] Papadakos JK, Hasan SM, Barnsley J, et al. Health literacy and cancer self-management behaviors: a scoping review. Cancer. 2018;124(21):4202–4210. doi:10.1002/cncr.31733. 30264856

[45] Nutbeam D, Lloyd JE. Understanding and responding to health literacy as a social determinant of health. Annu Rev Public Health. 2021;42(1):159–173. doi:10.1146/annurev-publhealth-090419-102529. 33035427

[46] Sørensen K, Levin-Zamir D, Duong TV, et al. Building health literacy system capacity: a framework for health literate systems. Health Promot Int. 2021;36(Suppl. 1):i13–i23. doi:10.1093/heapro/daab153. 34897445 PMC8672927

[47] Bröder J, Chang P, Kickbusch I, et al. IUHPE position statement on health literacy: a practical vision for a health literate world. Glob Health Promot. 2018;25(4):79–88. doi:10.1177/1757975918814421.

[48] Galea S, Tracy M. Participation rates in epidemiologic studies. Ann Epidemiol. 2007;17(9):643–653. doi:10.1016/j.annepidem.2007.03.013. 17553702

[49] Kripalani S, Goggins K, Couey C, et al. Disparities in research participation by level of health literacy. Mayo Clin Proc. 2021;96(2):314–321. doi:10.1016/j.mayocp.2020.06.058. 33549253 PMC7874435

[50] Sætre LB, Raasthøj I, Amalie S, et al. Exploring health literacy challenges in the general Danish population – a cross sectional study on the associations with individual factors and healthcare-seeking behaviour. Eur J Public Health. 2024. doi:10.1093/eurpub/ckae150 PMC11631385 39402975

[51] Osborne RH, Cheng CC, Nolte S, et al. Health literacy measurement: embracing diversity in a strengths-based approach to promote health and equity, and avoid epistemic injustice. BMJ Glob Health. 2022;7(9):e009623. doi:10.1136/bmjgh-2022-009623.

[52] Fortin M, Haggerty J, Sanche S, et al. Self-reported versus health administrative data: implications for assessing chronic illness burden in populations. A cross-sectional study. CMAJ Open. 2017;5(3):E729–E733. doi:10.9778/cmajo.20170029. PMC5621946 28947426

[53] Nicholson K, Almirall J, Fortin M. The measurement of multimorbidity. Health Psychol. 2019;38(9):783–790. doi:10.1037/hea0000739. 31021126

[54] Willadsen TG, Bebe A, Køster-Rasmussen R, et al. The role of diseases, risk factors and symptoms in the definition of multimorbidity – a systematic review. Scand J Prim Health Care. 2016;34(2):112–121. doi:10.3109/02813432.2016.1153242. 26954365 PMC4977932

[55] Huntley AL, Johnson R, Purdy S, et al. Measures of multimorbidity and morbidity burden for use in primary care and community settings: a systematic review and guide. Ann Fam Med. 2012;10(2):134–141. doi:10.1370/afm.1363. 22412005 PMC3315139

[56] Robinson KML, Jeppesen CJ, Vind M, et al. Kroniske sygdomme – hvordan opgøres kroniske sygdomme [Chronic diseases – how are chronic diseases measured]. Copenhagen: Forskningscenter for Forebyggelse og Sundhed; 2011.

[57] Ministry for the Interior and Health. Sundhedsstrukturkommissionens rapport. Report No. 978-87-7601-426-1; 2024.

