# Supplementary material for: Exploring perceived access to and previous experiences with general practice and associations with health literacy in the Danish population
Source: Scand J Prim Health Care. 2025 Nov 24;44(1):1–15. doi: 10.1080/02813432.2025.2583706 (PMC12918393; doi:10.1080/02813432.2025.2583706)
Supplement: SUPPLEMENTARY MATERIAL.docx [file IPRI_A_2583706_SM3468.docx]

| **SUPPLEMENTARY MATERIAL**  **Supplementary Table S1: Questionnaire and register data** | | |
| --- | --- | --- |
| ***Variable (s)*** | **Question (s)** | **Answer categories** |
| **Health Literacy Questionnaire*** | | |
| Feel understood and supported by healthcare providers  **“Supported and understood”** | I have at least one healthcare provider who knows me well…  I have at least one healthcare provider I can discuss…  I have the healthcare providers I need to help me work…  I can rely on at least one healthcare provider… | 1=strongly disagree, 2=disagree, 3=agree, 4= strongly agree |
| Have sufficient information to manage my health  **“Sufficient information”** | I feel I have good information about health…  I have enough information to help me deal with…  I am sure I have all the information I need to manage…  I have all the information I need to look after my health… | 1=strongly disagree, 2=disagree, 3=agree, 4= strongly agree |
| Have social support for health  **“Social support”** | I can get access to several people who understand and…  When I feel ill, the people around me really understand me...  If I need help, I have plenty of people I can rely on…  I have at least one person who can come to medical…  I have strong support from family and friends… | 1=strongly disagree, 2=disagree, 3=agree, 4= strongly agree |
| Ability to actively engage with healthcare providers  **“Actively engage”** | Make sure that healthcare providers understand your…  Feel able to discuss your health concerns with a…  Have good discussions about health with doctors…  Discuss things with healthcare providers until you understand…  Ask healthcare providers questions to get the… | 1=always difficult, 2=usually difficult, 3=sometimes difficult, 4=usually easy, 5=always easy |
| **Chronic disease** | Do you have any chronic disease, long-term effects after injuries, disability, or other chronic disorder? | Yes, No, I don’t know |
| **Register data** | | |
| **Covariate** | **Register** | **Categories** |
| **Sex** | Danish Civil Registration System | Civil Registration System Number  Ending on:   - equal number: females - unequal number: males |
| **Age** | Danish Civil Registration System | Civil Registration System Number   - counted at the time of invitation based on birthday. - Categorised as followed:   40-59 years  60-79 years  80+ years |
| **Marital status** | Danish Civil Registration System | Single/Living alone  Married/Living together |
| **Educational level** | Danish Education Register | Low: <10 years  Medium: 10-15 years  High: >15 years |
| **Labour market affiliation** | Income Statistics Register | Working  Pension  Out of workforce  Disability pension |
| **Ethnicity** | Danish Civil Registration System | Danish  Immigrants or descendants of immigrants |
| * The Health Literacy Questionnaire is under a licence, thus only fractions of the questions are allowed to be published | | |

| **Supplementary Table S2: Crude associations between sex, age, chronic disease and each of the seven statements regarding access, relationship and previous experiences with encounters in general practice (N=27,713)** | | | | | | | |
| --- | --- | --- | --- | --- | --- | --- | --- |
|  | **Access to general practice** | | | | | | |
|  | **Q1: Difficult to talk to the secretary (n=23,944)** | **Q2: Difficulty getting an appointment (n=25,995)** | | **Q3: Difficult to get in touch**  **(n=26,466)** | |  | |
|  | Cr. OR (95% CI) | Cr. OR (95% CI) | | Cr. OR (95% CI) | |  | |
| **Sex** |  |  | |  | |  | |
| Females | ref | ref | | ref | |  | |
| Males | **0.73(0.70-0.77)** | **0.70(0.67-0.74)** | | **0.72(0.69-0.76)** | |  | |
| **Age groups** |  |  | |  | |  | |
| 20-39 years | Ref | ref | | ref | |  | |
| 40-59 years | **0.90(0.84-0.97)** | **1.13(1.06-1.21)** | | **1.17(1.09-1.25)** | |  | |
| 60-79 years | **0.76(0.71-0.81)** | **0.72(0.67-0.77)** | | **0.78(0.73-0.83)** | |  | |
| 80+ years | **0.58(0.50-0.68)** | **0.49(0.42-0.56)** | | **0.58(0.50-0.67)** | |  | |
| **Chronic disease** |  |  | |  | |  | |
| No | ref | ref | | ref | |  | |
| Yes | **1.06(1.00-1.11)** | **1.05(1.00-1.10)** | | 1.02(0.97-1.07) | |  | |
|  | **Relationship with the general practitioner** | | | | **Experiences with previous encounters in general practice** | | |
|  | **Q4: Confident the doctor can help**  **(n=26,826)** | | **Q5: Like to be seen by the same doctor**  **(n=26,244)** | | **Q6: Negative experiences**  **(n=26,244)** | | **Q7: Too little time**  **(n=26,300)** |
|  | Cr. OR (95% CI) | | Cr. OR (95% CI) | | Cr. OR (95% CI) | | Cr. OR (95% CI) |
| **Sex** |  | |  | |  | |  |
| Females | ref | | ref | | ref | | ref |
| Males | 1.04(0.98-1.11) | | **0.76(0.71-0.81)** | | **0.59(0.56-0.62)** | | 0.65(0.62-0.69) |
| **Age groups** |  | |  | |  | |  |
| 20-39 years | ref | | ref | | ref | | ref |
| 40-59 years | **1.12(1.03-1.22)** | | **1.38(1.26-1.50)** | | **0.74(0.69-0.79)** | | **1.10(1.03-1.18)** |
| 60-79 years | 1.06(0.97-1.15) | | **1.42(1.30-1.55)** | | **0.47(0.44-0.50)** | | **0.80(0.75-0.86)** |
| 80+ years | **0.81(0.69-0.95)** | | **1.24(1.05-1.48)** | | **0.33(0.28-0.39)** | | **0.57(0.50-0.65)** |
| **Chronic disease** |  | |  | |  | |  |
| No | ref | | ref | | ref | | ref |
| Yes | 0.85(0.80-0.90) | | **1.20(1.12-1.29)** | | **1.45(1.38-1.52)** | | **1.30(1.24-1.37)** |
| **Bold** statistically significant, p-value< 0.05, Cr.=crude, OR = odds ratio, CI = confidence interval | | | | | | | |

| **Supplementary Table S3: Crude associations between health literacy and each of the seven statements regarding access, relationship and previous experiences with encounters in general practice (N=27,713)** | | | | | | | | | |
| --- | --- | --- | --- | --- | --- | --- | --- | --- | --- |
|  | **Access to general practice** | | | | | |  | | |
|  | **Q1: Difficult to talk to the secretary (n=23,944)** | | **Q2: Difficulty getting an appointment (n=25,995)** | | **Q3: Difficult to get in touch**  **(n=26,466)** | |  | |  |
|  | Cr. OR (95% CI) | | Cr. OR (95% CI) | Adj.* OR (95% CI) | Cr. OR (95% CI) | Adj.* OR (95% CI) |  |  |  |
| **Health literacy** |  |  |  |  |  |  |  |  |  |
| ’Understood and supported’ | **0.52(0.50-0.55)** | | **0.47(0.46-0.49)** | | **0.44(0.42-0.46)** | |  |  |  |
| ’Sufficient information’ | **0.49(0.47-0.52)** | | **0.54(0.52-0.57)** | | **0.52(0.50-0.55)** | |  |  |  |
| ’Social support’ | **0.54(0.52-0.57)** | | **0.62(0.60-0.65)** | | **0.60(0.58-0.63)** | |  |  |  |
| ’Actively engage’ | **0.42(0.41-0.44)** | | **0.50(0.48-0.51)** | | **0.47(0.46-0.49)** | |  |  |  |
|  | **Relationship with the general practitioner** | | | | **Experiences with previous encounters in general practice** | | | | |
|  | **Q4: Confident the doctor can help**  **(n=26,826)** | | **Q5: Like to be seen by the same doctor**  **(n=26,244)** | | **Q6: Negative experiences**  **(n=26,244)** | | **Q7: Too little time**  **(n=26,300)** | |  |
|  | Cr. OR (95% CI) | | Cr. OR (95% CI) | | Cr. OR (95% CI) | | Cr. OR (95% CI) | |  |
| **Health literacy** |  |  |  |  |  |  |  |  |  |
| ’Understood and supported’ | **2.01(1.92-2.11)** | | **1.11(1.06-1.17)** | | **0.46(0.44-0.47)** | | **0.47(0.45-0.49)** | |  |
| ’Sufficient information’ | **1.85(1.75-1.95)** | | **0.97(0.91-1.02)** | | **0.48(0.46-0.50)** | | **0.49(0.47-0.51)** | |  |
| ’Social support’ | **1.65(1.56-1.73)** | | **0.97(0.92-1.03)** | | **0.57(0.54-0.59)** | | **0.56(0.54-0.58)** | |  |
| ’Actively engage’ | **1.74(1.68-1.80)** | | **0.94(0.90-0.97)** | | **0.38(0.37-0.39)** | | **0.40(0.38-0.41)** | |  |
| **Bold** statistically significant, *p*-value< 0.05  Cr.=crude, OR = odds ratio, CI = confidence interval | | | | | | | | | |

| **Supplementary Table S4: Crude associations between socioeconomics and each of the seven statements regarding access, relationship and previous experiences with encounters in general practice (N=27,713)** | | | | | | | | |
| --- | --- | --- | --- | --- | --- | --- | --- | --- |
|  | **Access to general practice** | | | | | |  | |
|  | **Q1: Difficult to talk to the secretary (n=23,944)** | | **Q2: Difficulty getting an appointment (n=25,995)** | | **Q3: Difficult to get in touch**  **(n=26,466)** | |  | |
|  | Agree, n (%) | Cr. OR (95% CI) | Agree, n (%) | Cr. OR (95% CI) | Agree, n (%) | Cr. OR (95% CI) |  |  |
| **Marital status** |  |  |  |  |  |  |  |  |
| Single/living alone | 5996(43.4) | ref | 7608(51.1) | ref | 10777(40.7) | ref |  |  |
| Married/living together | 3660(36.1) | **0.93(0.88-0.99)** | 4687(42.2) | **1.05(1.00-1.11)** | 6672(44.0) | **1.08(1.02-1.14)** |  |  |
| **Highest obtained level of education** |  |  |  |  |  |  |  |  |
| Low (<10 years) | 2238(44.2) | ref | 2611(50.1) | ref | 2250(42.1) | ref |  |  |
| Middle (10-15 years)) | 3758(41.8) | **0.90(0.81-0.99)** | 5089(53.2) | **1.20(1.08-1.32)** | 4478(46.0) | **1.10(1.00-1.22)** |  |  |
| High (>15 years) | 3355(37.6) | **0.77(0.70-0.86)** | 4238(41.8) | **1.29(1.16-1.42)** | 3722(36.2) | **1.11(1.00-1.22)** |  |  |
| **Labour market affiliation** |  |  |  |  |  |  |  |  |
| Working | 305(31.6) | ref | 357(32.8) | ref | 327(29.6) | ref |  |  |
| Pension | 5386(39.7) | **0.79(0.74-0.84)** | 6872(46.8) | **0.61(0.57-0.64)** | 6082(40.6) | **0.66(0.62-0.70)** |  |  |
| Out of workforce | 4270(41.1) | **1.25(1.13-1.39)** | 5423(48.0) | 0.91(0.82-1.00) | 4695(41.0) | **0.88(0.80-0.97)** |  |  |
| Disability pension | 5996(43.4) | **1.34(1.17-1.53)** | 7608(51.1) | 0.89(0.78-1.01) | 10777(40.7) | 0.92(0.81-1.05) |  |  |
| **Ethnicity** |  |  |  |  |  |  |  |  |
| Danish | 3660(36.1) | ref | 4687(42.2) | ref | 6672(44.0) | ref |  |  |
| Immigrants/descendants of immigrants | 2238(44.2) | **1.39(1.26-1.53)** | 2611(50.1) | 1.08(0.98-1.19) | 2250(42.1) | 1.03(0.93-1.13) |  |  |
|  | **Relationship with the general practitioner** | | | | **Experiences with previous encounters in general practice** | | | |
|  | **Q4: Confident the doctor can help**  **(n=26,826)** | | **Q5: Like to be seen by the same doctor**  **(n=26,244)** | | **Q6: Negative experiences**  **(n=26,244)** | | **Q7: Too little time**  **(n=26,300)** | |
|  | Agree, n (%) | Cr. OR (95% CI) | Agree, n (%) | Cr. OR (95% CI) | Agree, n (%) | Cr. OR (95% CI) | Agree, n (%) | Cr. OR (95% CI) |
| **Marital status** |  |  |  |  |  |  |  |  |
| Single/living alone | 12550(81.8) | ref | 12865(85.6) | ref | 6013(40.1) | ref | 7941(52.7) | ref |
| Married/living together | 9477(82.4) | **1.22(1.14-1.31)** | 9178(81.9) | **1.09(1.01-1.17)** | 3177(28.2) | **0.82(0.78-0.87)** | 4733(42.1) | **0.92(0.87-0.97)** |
| **Highest obtained level of education** |  |  |  |  |  |  |  |  |
| Low (<10 years) | 4366(81.3) | ref | 4148(80.2) | ref | 2429(45.4) | ref | 2634(50.0) | ref |
| Middle (10-15 years) | 8175(83.0) | **1.26(1.12-1.42)** | 8154(84.8) | 1.12(0.98-1.27) | 3693(38.0) | **1.14(1.02-1.27)** | 5085(52.4) | **1.14(1.04-1.26)** |
| High (>15 years) | 8604(82.2) | **1.36(1.21-1.54)** | 8806(85.2) | 1.09(0.95-1.24) | 2839(28.1) | **1.17(1.05-1.30)** | 4556(44.5) | 1.07(0.97-1.18) |
| **Labour market affiliation** |  |  |  |  |  |  |  |  |
| Working | 882(77.8) | ref | 935(83.5) | ref | 229(21.6) | ref | 399(36.3) | ref |
| Pension | 12700(83.2) | **0.86(0.80-0.93)** | 12360(83.0) | **1.14(1.06-1.24)** | 4692(31.4) | **0.61(0.57-0.65)** | 6746(45.3) | 0.71(0.67-0.75) |
| Out of workforce | 9327(80.7) | **0.73(0.64-0.82)** | 9683(85.4) | 1.09(0.95-1.25) | 4498(39.8) | **1.63(1.47-1.79)** | 5928(51.9) | **1.21(1.10-1.34)** |
| Disability pension | 12550(81.8) | **0.65(0.56-0.76)** | 12865(85.6) | 1.04(0.87-1.24) | 6013(40.1) | **1.65(1.45-1.88)** | 7941(52.7) | **1.30(1.14-1.48)** |
| **Ethnicity** | 9477(82.4) |  | 9178(81.9) |  | 3177(28.2) |  | 4733(42.1) |  |
| Danish |  | ref |  | ref |  | ref |  | ref |
| Immigrants/descendants of immigrants | 4366(81.3) | **0.70(0.62-0.78)** | 4148(80.2) | **0.77(0.68-0.87)** | 2429(45.4) | 1.08(0.98-1.19) | 2634(50.0) | **1.27(1.15-1.39)** |
| **Bold** statistically significant, *p*-value< 0.05, Cr.=crude, OR = odds ratio, CI = confidence interval | | | | | | | | |
